# Lichen planus with multiple system involvement including the mouth, vagina, urethra, and scalp: a case report

**DOI:** 10.1590/1414-431X20198823

**Published:** 2019-10-10

**Authors:** Lan Mi, Hong Zhang, Dai Zhang, Miao Zhang

**Affiliations:** 1 Department of Obstetrics and Gynecology, Peking University First Hospital, Beijing, China; 2 Department of Pathology, Peking University First Hospital, Beijing, China; 3 Department of Obstetrics and Gynecology, Beijing Aiyuhua Hospital for Women and Children, Beijing, China

**Keywords:** Lichen planus, Intercourse pain, Multiple system involvement, Corticosteroid, Immunosuppressant, Case report

## Abstract

This is a case report of lichen planus (LP) with multiple system involvement. A 35-year-old female patient was admitted in November 2014 with a 5-year history of painful/difficult sexual intercourse and loss of oral mucosa, and an 8-year history of focal hair loss. Earlier, the patient had been unable to adhere to corticosteroid therapy because of severe adverse side effects. In September 2014, labia minora mucosa defects and stricture of the urethral orifice (with dysuria), vaginal orifice, and vagina were identified. Biopsy was performed and a diagnosis of erosive LP was made. The patient was treated with an oral immunosuppressant (cyclosporine A) and urethral/vaginal dilatation. Urine flow rate and sex life were improved after 6 months and she discontinued medication. Four years later, the patient reported a good overall treatment efficacy. LP can involve multiple systems and should be considered in patients with dyspareunia. Immunosuppressive agents can achieve a satisfactory effect in patients with contraindication to corticosteroid.

## Introduction

Lichen planus (LP) is a relatively rare chronic inflammatory skin disease. Epidemiological data about LP are limited, but a review of 45 studies showed that the prevalence of oral LP is between 1 and 3%; only one study could calculate the effective age-standardized prevalence rate, which was 1.57% in women (1). LP is more common in women aged 50–60 years than in women of childbearing age. LP can present with extensive involvement of the skin, mucosa, nails, and scalp, but it can also occur only in the vulva. Vulvar LP (VLP) is a subtype of LP characterized by erosive, papular, or hypertrophic vulvar lesions with or without vaginal involvement. The exact incidence of VLP is currently unclear. Studies based on outpatient biopsy of the vulva have reported a VLP incidence of 3.7% (2), but up to 57% of patients with oral LP also have VLP (3). Clinical manifestations of VLP include irritating vaginal discharge, vulvar tenderness, severe itching, burning sensation, and pain on intercourse, although a small number of patients are asymptomatic or have only mild symptoms (4). Currently, there are no evidence-based management guidelines for VLP (4). Furthermore, there are no published studies of high quality evaluating the treatment of LP. Most clinicians consider topical corticosteroids as the first-line therapy for VLP, and vaginal corticosteroids are given as a first-line therapy for patients with vaginal involvement (5). This case report describes the diagnosis and treatment of LP with multiple system involvement in a Chinese woman of childbearing age.

## Case Report

**Figure 1.**
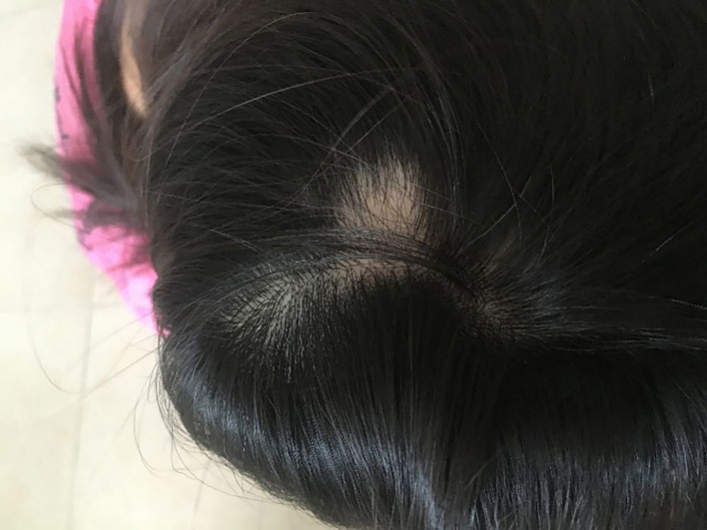
Zone of hair loss.

The patient provided informed consent for inclusion in this case report, and Peking University First Hospital committee approved the study. A 35-year-old female patient of Han ethnicity was admitted to our hospital with a 5-year history of painful intercourse accompanied by oral mucosal lesions and a 3-year history of difficulty with intercourse. The patient began a normal sex life in 2005 and in the same year had a medical abortion. In 2006, the patient experienced partial hair loss over an area ∼1 cm in diameter on the head (Figure 1). The patient considered the hair loss to be alopecia areata and did not consult a doctor. In January 2007, a medical abortion at 16 weeks of pregnancy was performed, and the fetus and placenta were successfully delivered; uterine curettage was not performed. The patient recovered her menstrual cycle after one month and resumed normal sexual activity after 2 months.

In 2009, the patient started experiencing symptoms of vaginal dryness and deep dyspareunia. In June 2009, the patient experienced gingival pain after eating spicy food and subsequently developed bilateral oral mucosal peeling, but she did not consult a doctor. In 2010, the patient was admitted to another hospital due to aggravation of the symptoms of vaginal dryness during sexual intercourse and the development of pain. A vaginal mucosal ulcer with a diameter of about 8 mm was found. The patient was admitted to Union Medical College Hospital, where tests for anti-nuclear antibody spectrum and lupus antibody were normal, as were the results of other examinations. Behcet's disease was excluded, and she did not receive a formal diagnosis or any treatment.

**Figure 2.**
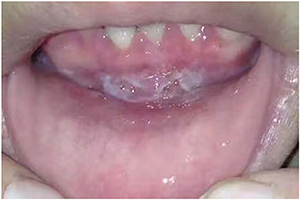
Ulceration of the oral mucosa.

In 2011, the patient developed difficulty having sexual intercourse, and the main manifestation was pain at the vaginal orifice that prevented penetration. The patient had no sex life after 2013. In January 2014, the patient was admitted to the Stomatological Hospital, and physical examination showed that the oral lesions (Figure 2) resembled LP. Other than the oral ulcers and hyperemia, there was no symptoms in other parts of the oral cavity (gingiva or throat). She was treated with oral prednisone (4 mg qd), *Tripterygium wilfordii*, mouthwash (containing 5 mg/mL dexamethasone sodium phosphate 1 mL twice a day), and thalidomide. The patient reported increased nocturia and vivid dreams during the treatment period, and she refused to continue the therapy. She had a history of trichomonas vaginitis twice in 2013.

A vaginal microecological examination in August 2014 showed an abnormal flora, and a lactobacillus capsule was administered to the upper vagina once daily for 10 days. The patient was previously healthy (before the onset of symptoms) and denied a history of surgery. The menstrual cycle was 4/25–28 days, and menstrual volume had decreased during the previous year, but there was no dysmenorrhea. The last menstrual period had occurred on September 12, 2014. The patient was gravida 2 (one medical abortion and one induction of labor in the second trimester), para 0. She had an allergy to penicillin. There was no relevant family history of note. The patient's husband was 34 years old and had normal erectile function.

**Figure 3.**
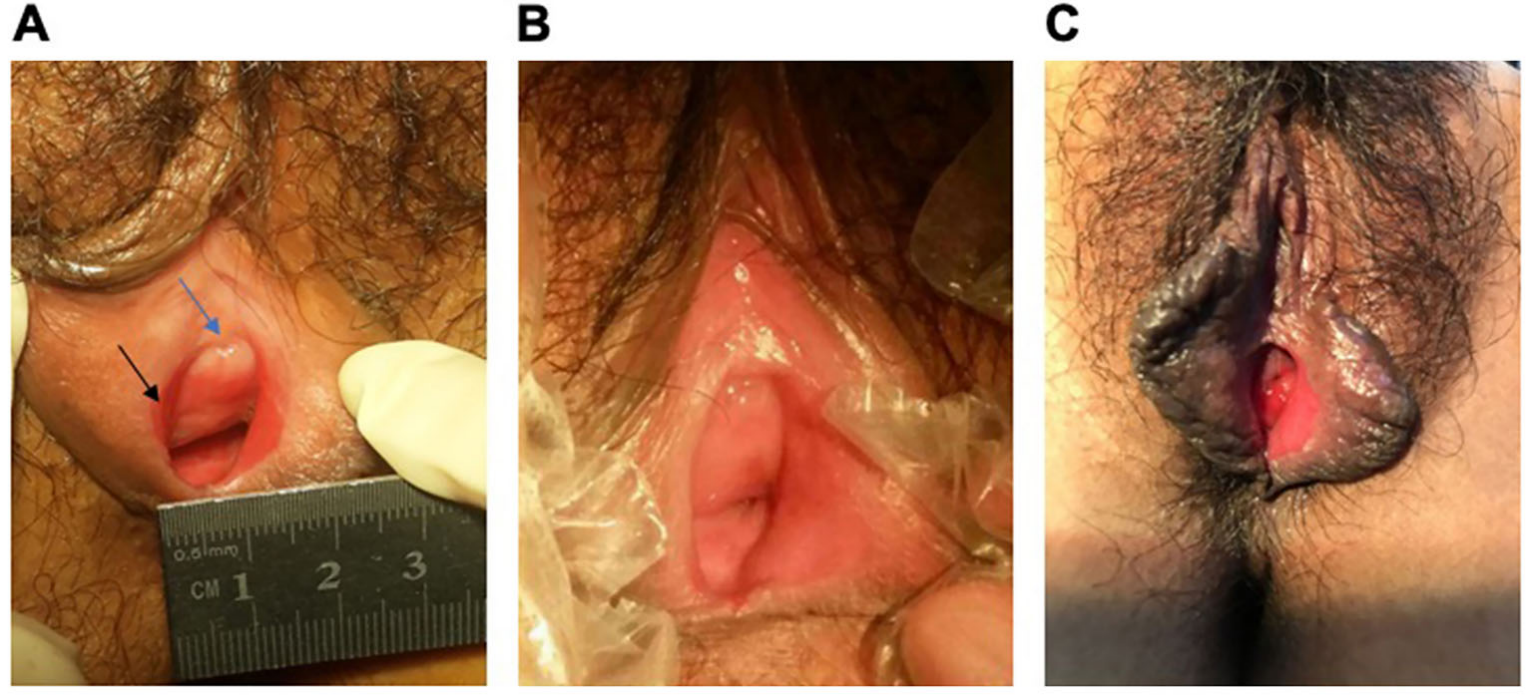
**A**, The medial mucosal surfaces of the labia minora had bright red-colored, defect-like lesions, and the vaginal orifice was stenosed with a diameter of 1 cm. Blue arrow: narrow urethral orifice; Black arrow: defect of the mucosa. **B**, The medial mucosa of the labia minora was smooth and slightly reddened, and the vaginal opening was slightly narrowed. **C**, The mucosa of the medial labia minora was slightly reddened, smooth, and without defects.

In September 2014, the patient was admitted to the Department of Obstetrics and Gynecology, Peking University First Hospital, due to inability to have sexual intercourse. She had a height of 155 cm and a weight of 46.5 kg. Examination of the vulva revealed an extremely thin mucous membrane, hyperemia, a slight lowering of the pubic arch, and a small, bright-red, defect-like lesion on the mucosal surface of the medial labia minora. The vaginal orifice was stenosed with a diameter of 1 cm. There was vaginal stenosis with disappearance of the mucosal folds. The urethral orifice was less than 1 mm in diameter. Ring-like stenosis was present at the 9 o'clock position of the upper two-thirds of the vagina. A small speculum was unable to expose the cervix (Figure 3A). The pH of the vaginal secretions was 5.4. Hormone levels were normal (luteinizing hormone, 4.92 mIU/mL; follicle stimulating hormone, 8.12 mIU/mL; estradiol, 43.0 pg/mL; prolactin, 26.3 ng/mL; and testosterone, 0.48 ng/mL). Tests for autoimmune antibodies (anti-nuclear antibody, anti-double-stranded DNA antibody, and anti-extractable nuclear antigen) were negative, and tests for anti-thyroid antibodies were also normal (anti-thyroperoxidase antibody, 16.06 IU/mL; anti-thyroglobulin antibody, 23.05 IU/mL). Examination of the eyes showed no abnormalities.

**Figure 4.**
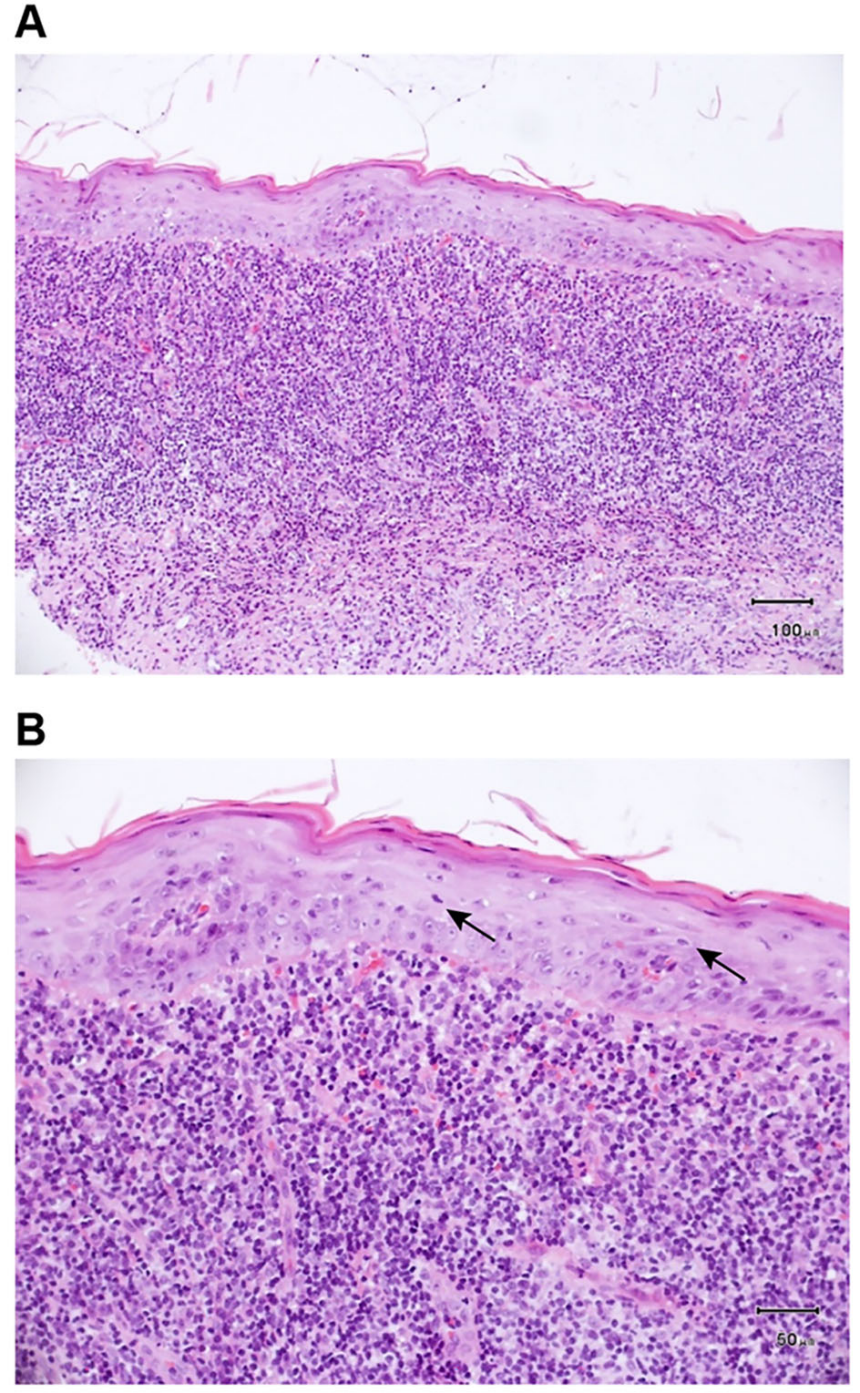
Biopsy of right buccal mucosa (taken beside the ulcer) showing squamous epithelial mucosa and squamous epithelial dyskeratosis. **A**, Superficial zonular lymphocytes and plasma cells were distributed in the lamina propria, and there was small vessel hyperplasia. Scale bar: 100 μm. **B**, Lymphocytes were scattered within the epithelium (black arrows). Scale bar: 50 μm.

An oral mucosal biopsy (performed due to the history of oral mucosal ulceration) revealed the following results for the right cheek: squamous epithelial mucosa, squamous epithelial parakeratosis, scattered lymphocytes in the epithelium, lymphoid and plasma cells distributed in a strip-like pattern in the superficial lamina propria, and small vessel hyperplasia (Figure 4A and B).

**Figure 5.**
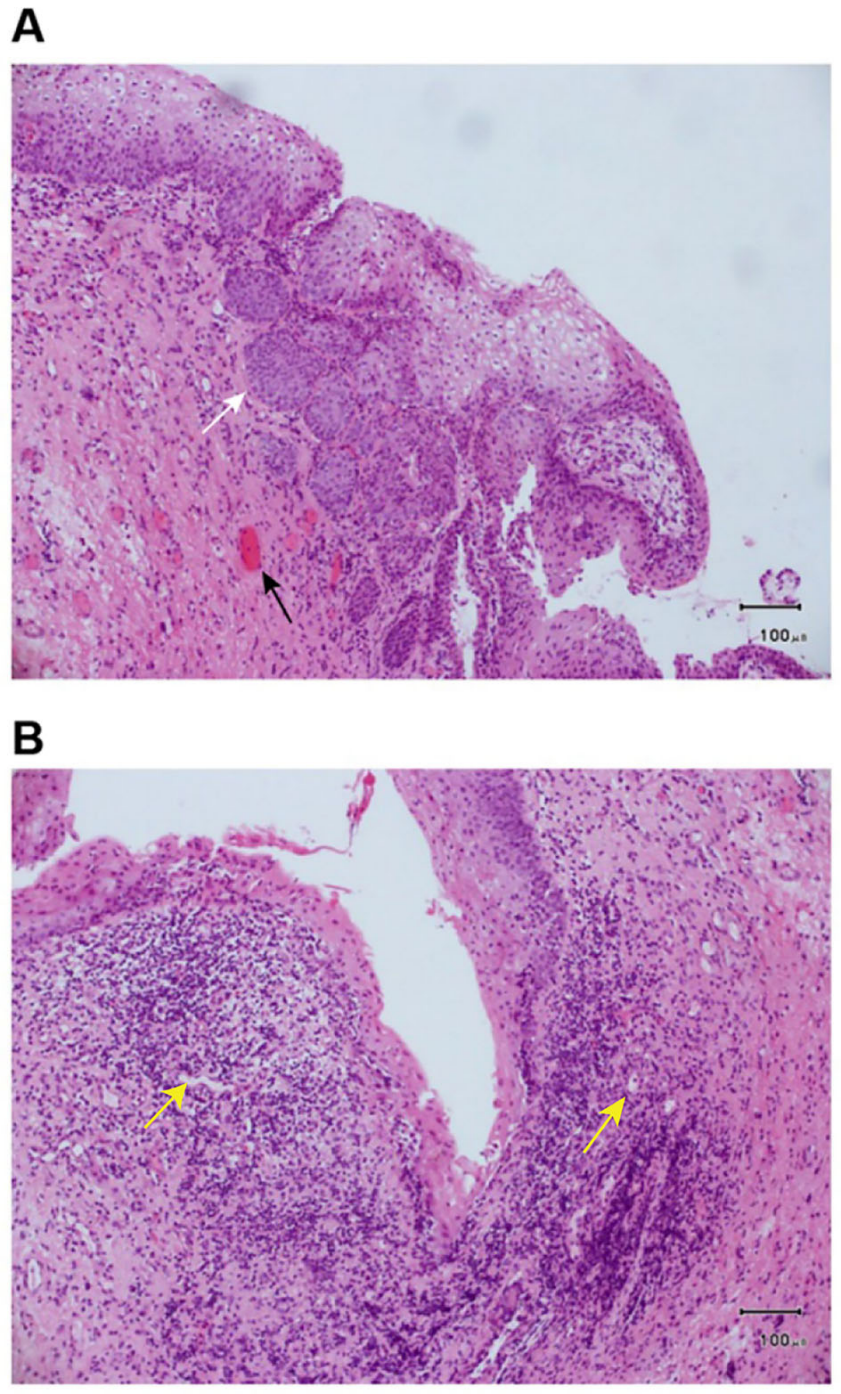
Vaginal wall biopsy. **A**, Squamous epithelial mucosa, with simple and pseudoepithelioma-like hyperplasia (white arrow) in some epithelium, hyperkeratosis and incomplete keratosis, and hyperplasia of interstitial small vessels (black arrow) Scale bar: 100 μm. **B**, Squamous epithelial mucosa, atrophy of part of the squamous epithelium, scattered superficial dermis, infiltration of focal lymph and plasma cells, and formation of lymphoid follicles (yellow arrows). Scale bar: 100 μm.

A vaginal wall biopsy was performed on November 17, 2014 after preoperative tests. The pathological sections were considered to have squamous epithelial mucosal tissue, partial epithelial atrophy, partial simple hyperplasia, hyperkeratosis and incomplete keratosis, scattered focal infiltration of lymphocytes and plasma cells in the superficial dermis with formation of lymphoid follicles, and small vessel hyperplasia (Figure 5A and B). These results were consistent with an inflammatory lesion.

**Figure 6.**
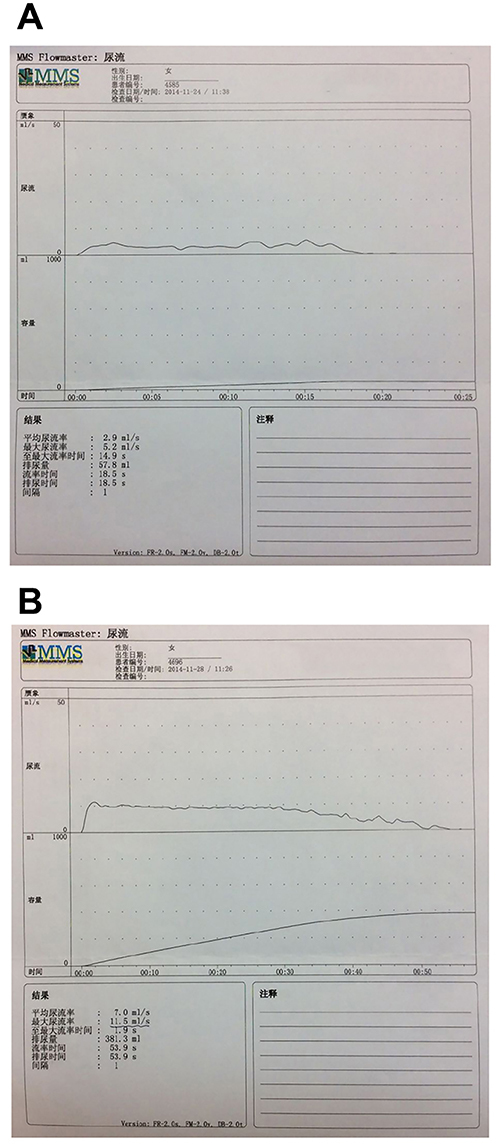
Measurement of urine flow rate. **A**, A maximum urine flow rate of 5.2 mL/s was measured before treatment with urethral dilatation and oral cyclosporine A. **B**, A maximum urine flow rate of 11.5 mL/s was measured after treatment with urethral dilatation and oral cyclosporine A.

In view of the symptoms of urinary difficulty and the discovery of a pinpoint urethral orifice on examination, urine flow rate was measured at the Urology Department and found to be 5.2 mL/s (normal value: >18 mL/s) (Figure 6A).

After multidisciplinary discussion of the case between the Departments of Dermatology, Obstetrics and Gynecology, and Urology at our hospital, the patient was diagnosed as having LP with multiple system involvement including the scalp, mouth, vagina, and urethra. Since the patient had not tolerated the adverse side effects of corticosteroid therapy during previous treatment and had fertility requirements, she was treated with oral cyclosporine A (125 mg/day) combined with physical dilatation of the urethra, and this resulted in an increase in urine flow rate to 11.5 mL/s (Figure 6B).

After one month of drug therapy (January 8, 2015), the patient resumed her sex life and had sexual intercourse for 10 min without serious dyspareunia. In addition, the oral ulcer had improved, and urination was less problematic than before treatment but more difficult than just after urethral dilatation. Physical examination revealed that the mucosa of the labia minora was smooth, the frenulum labiorum pudendi was lightly chapped and the vaginal elasticity had improved, although the upper vagina still had a ring-like narrowing and no folds (Figure 3B).

At the 3-month follow-up, the patient reported only mild pain during sexual activity, but a feeling of dryness persisted. There was no change in urination, and no oral mucosal lesions were present. The patient was treated with cyclosporine A for a total of 6 months, and this resulted in the vulvar lesions essentially disappearing (as assessed by gynecological examination), although the symptoms did not notably improve during the latter 3 months. The patient discontinued the medication due to economic pressures.

The patient was followed-up annually with gynecological examinations for a further 4 years. During the 4-year follow-up period, the patient reported that her psychological stress had been relieved and that symptom aggravation had not occurred. In July 2018 (the last outpatient follow-up), the patient reported a satisfactory sex life and a slight decrease in urine flow rate, but no treatment was needed. Oral mucosal lesions had not re-appeared. Gynecological examination showed that the mucosa of the medial labia minora was slightly reddened, smooth, and without defects, the elasticity of the vaginal mucosa was good, and two fingers could be accommodated (see Figure 3C). There was no discomfort during the physical examinations. At present, the patient is not pregnant and continues to be followed-up.

## Discussion

LP is rare in women of childbearing age and has a low incidence of multiple-system involvement. A review showed that the prevalence of oral LP is between 1 and 3% (1), while a study reported a prevalence of about 0.03% in children (6). Cassol-Spanemberg et al. (7) retrospectively studied 274 patients with histologically confirmed oral LP and found 21 (7.7%) patients with genital involvement. Saunders et al. (8) reported 13 cases of coexisting oral LP and vulvar lichen sclerosus. At their center and during their study period, there were six cases of LP per 1000 cases of vulvar LP (8). Petruzzi et al. (9) reported two cases of LP of the oral cavity, vagina, and vulva.

The primary manifestation of this case of LP was the involvement of multiple systems, including the mouth, scalp, vagina, and urethra. The main gynecological symptom was dyspareunia, and vulvar lesions were not obvious. Dyspareunia is usually primary and related to intercourse pain and vaginal spasms, whereas secondary dyspareunia usually results from organic diseases, drug effects, or psychiatric disorders. The patient initially had a normal sex life and underwent a mid-term medical abortion, which excluded primary intercourse pain and vaginal spasms. Furthermore, the patient had no history of taking medications that might have led to secondary dyspareunia. Although she was under psychological stress due to her family not approving of her relationship, the patient did not have a psychiatric illness. Thus, we concluded that an organic disease was the most likely cause based on the history and symptoms.

The relevant hospital departments were then asked to formally evaluate the involvement of each system, and the results of oral and vaginal mucosal biopsies and urinary flow rate measurement were considered. After multidisciplinary consultation among the Dermatology, Urology, and Obstetrics and Gynecology Departments of our hospital, a diagnosis was made of LP with multiple system involvement.

The etiology of LP is not fully understood, and the characteristic lesion of VLP is thought to be caused by a T cell-mediated autoimmune response against basal keratinocytes (10). The existence of rare cases of familial LP and the overexpression of certain HLA haplotypes (such as HLA-DR1 in skin LP) suggest that genetic factors have an impact on the susceptibility to this disease (11). At present, LP is believed to be caused by multiple factors, of which autoimmunity is the most relevant (12). The patient had no basis for an autoimmune disease, so the cause of the disease was unknown. The average age of onset of VLP is 50–60 years, but the patient was only 30 years old at the time of onset, suggesting that VLP should be considered in young women with secondary dyspareunia.

There are four types of LP involving the vulva, including erosive LP, papular squamous LP, hypertrophic LP, and hairy LP (13). Erosive LP is the most common type of vulvar LP encountered in secondary medical institutions. This desquamate, erosive chronic dermatitis often affects the vagina, with repeated episodes followed by slow healing sometimes resulting in scarring. Scar formation can lead to extensive anatomical damage, including severe vaginal stenosis and urethral obstruction. Painful intercourse, intercourse failure, dysuria, and urodynia are the main symptoms of erosive LP. The findings in this patient on admission to our hospital were typical of erosive LP, with ring stenosis at the 9 o'clock position of the upper two-thirds of the vagina and urethral stricture thought to be due to scar formation.

A retrospective analysis of 100 consecutive patients diagnosed with VLP at the Mayo Clinic between 1997 and 2015 found that 46% of the patients were diagnosed within 1 year and 36% after more than 4 years of symptom onset. On review of the medical history of the patient in this case report, it was evident that the symptoms had started a long time previously and gradually worsened, but a clear diagnosis had not been reached despite multiple visits to different departments. The patient was finally diagnosed after a multidisciplinary consultation at our hospital.

In a study by Cassol-Spanemberg et al. (7), six patients first developed LP of the genital organs, but had no oral symptoms. They suggested that gynecologists refer the patients to the stomatology department to look for LP of the oral cavity. It has been suggested that gynecologists should consider this disease when encountering patients with dysphagia and actively contact the dermatology and other departments for multidisciplinary discussion (4). It has been reported that vaginal involvement occurs in up to 70% of patients with erosive LP but is rare in sclerosing LP (14), hence these two conditions can be distinguished. Vulvo-vaginal-gingival syndrome has gingival involvement and is especially resistant to treatment. The oral ulcer of this patient was entirely within the buccal mucosa with no gingival involvement, excluding vulvo-vaginal-gingival syndrome.

Treatment of LP includes drug therapy and adjuvant therapy. At present, there are no guidelines for medical treatment, and data are limited regarding the management of VLP (15). So far, only one randomized controlled trial has evaluated the treatment of vulvar erosive LP (16). The treatment methods currently used are mainly based on studies of case series and reports of clinical experience.

Topical corticosteroids are recognized as a first-line treatment for LP. In a study of 114 patients with biopsy-diagnosed VLP, 89 patients used super-potent topical steroids as a first-line treatment: the symptoms improved in 84 patients (94%) and disappeared in 63 patients (71%) (5). A typical treatment regimen involves the application of half a fingertip unit of a super-potent topical corticosteroid (for example, 0.05% clobetasol propionate ointment or 0.05% halobetasol propionate ointment) on the affected site once per night. Subsequent evaluations are performed after 2–3 months, and maintenance treatment is undertaken if the symptoms have been relieved and all the erosions have healed. A second-line treatment is topical tacrolimus (a calcineurin inhibitor), but high-quality data are lacking regarding the efficacy of this agent in the treatment of LP. In a retrospective study of 16 women with VLP, all but one of the patients had responded to topical tacrolimus with improvements of the lesions after 3 months of therapy (17). Systemic medication can be given if first-line and second-line treatments are ineffective and an aggravating factor for symptom persistence is not identified.

A variety of systemic immunomodulators and immunosuppressive agents have been used in individual patients (e.g., methotrexate, mycophenolate mofetil, oral or intramuscular injections of glucocorticoids, hydroxychloroquine, acitretin, minocycline, and cyclosporine) (18–20). However, the currently available data are not sufficient to confirm the efficacy of these specific interventions.

The patient in this case report was intolerant to the adverse side effects of corticosteroid therapy and so was unable to receive systemic or local corticosteroid therapy. Since economic factors precluded the use of tacrolimus, cyclosporine A was selected as the treatment of choice, and the effect was satisfactory. Adjuvant therapy includes patient education, vulvar care, and a review of medication history.

LP is a chronic disease; the lesions show delayed healing, and the symptoms can easily relapse after remission. Patients should be informed about the characteristics of the disease so that their expectations are reasonable. In addition, patients should be followed-up regularly, enabling timely intervention if relapse occurs. In addition, attention should be paid to the patient's psychological state and living habits. Because LP affects quality of life, psychological counseling can help to relieve stress. During follow-up, the patient should also be informed about the correct methods for perineal care, as this can facilitate physical and mental recovery.

Organic diseases can be secondary causes of difficulties with sexual intercourse, and LP with vaginal involvement is among the common causes. LP can affect the scalp, oral cavity, urethra, vagina, vulva, and other systems. The diagnosis of this disease requires consideration of the patient's medical history, and typical symptoms and signs.

The results of this study showed that immunosuppressive agents can achieve satisfactory therapeutic effects in patients intolerant to corticosteroid therapy. Long-term follow-up and counseling for psychological stress can help to prevent relapse.

## References

[1] McCartan BE, Healy CM (2008). The reported prevalence of oral lichen planus: a review and critique. J Oral Pathol Med.

[2] Micheletti L, Preti M, Bogliatto F, Zanotto-Valentino MC, Ghiringhello B, Massobrio M (2000). Vulval lichen planus in the practice of a vulval clinic. Br J Dermatol.

[3] Belfiore P, Di Fede O, Cabibi D, Campisi G, Amaru GS, De Cantis S (2006). Prevalence of vulval lichen planus in a cohort of women with oral lichen planus: an interdisciplinary study. Br J Dermatol.

[4] Fahy CMR, Torgerson RR, Davis MDP (2017). Lichen planus affecting the female genitalia: a retrospective review of patients at Mayo Clinic. J Am Acad Dermatol.

[5] Cooper SM, Haefner HK, Abrahams-Gessel S, Margesson LJ (2008). Vulvovaginal lichen planus treatment: a survey of current practices. Arch Dermatol.

[6] Pendyala G, Joshi S, Kalburge J, Joshi M, Tejnani A (2012). Oral lichen planus: a report and review of an autoimmune-mediated condition in gingiva. Compend Contin Educ Dent.

[7] Cassol-Spanemberg J, Blanco-Carrion A, Rodriguez-de Rivera-Campillo ME, Estrugo-Devesa A, Jane-Salas E, Lopez-Lopez J (2019). Cutaneous, genital and oral lichen planus: a descriptive study of 274 patients. Med Oral Patol Oral Cir Bucal.

[8] Saunders H, Buchanan JA, Cooper S, Hollowood K, Sherman V, Wojnarowska F (2010). The period prevalence of oral lichen planus in a cohort of patients with vulvar lichen sclerosus. J Eur Acad Dermatol Venereol.

[9] Petruzzi M, De Benedittis M, Carriero C, Giardina C, Parisi G, Serpico R (2005). Oro-vaginal-vulvar lichen planus: report of two new cases. Maturitas.

[10] Pittelkow MR, Daoud MS (2008). Lichen planus.

[11] Lewis FM (1998). Vulval lichen planus. Br J Dermatol.

[12] Cooper SM, Ali I, Baldo M, Wojnarowska F (2008). The association of lichen sclerosus and erosive lichen planus of the vulva with autoimmune disease: a case-control study. Arch Dermatol.

[13] Neill SM (1999). Erosive lichen planus: diagnosis and management. Syllabus of the post-graduate course, International Society for the Study of Vulvovaginal Disease.

[14] Zendell K, Edwards L (2013). Lichen sclerosus with vaginal involvement: report of 2 cases and review of the literature. JAMA Dermatol.

[15] Cheng S, Kirtschig G, Cooper S, Thornhill M, Leonardi-Bee J, Murphy R (2012). Interventions for erosive lichen planus affecting mucosal sites. Cochrane Database Syst Rev.

[16] Helgesen AL, Warloe T, Pripp AH, Kirschner R, Peng Q, Tanbo T (2015). Vulvovaginal photodynamic therapy vs. topical corticosteroids in genital erosive lichen planus: a randomized controlled trial. Br J Dermatol.

[17] Byrd JA, Davis MD, Rogers RS (2004). Recalcitrant symptomatic vulvar lichen planus: response to topical tacrolimus. Arch Dermatol.

[18] Bradford J, Fischer G (2013). Management of vulvovaginal lichen planus: a new approach. J Low Genit Tract Dis.

[B19] Simpson RC, Littlewood SM, Cooper SM, Cruickshank ME, Green CM, Derrick E (2012). Real-life experience of managing vulval erosive lichen planus: a case-based review and U.K. multicentre case note audit. Br J Dermatol.

[20] Deen K, McMeniman E (2015). Mycophenolate mofetil in erosive genital lichen planus: a case and review of the literature. J Dermatol.

